# Abstracts From the First Annual International Infectious Disease Society for Obstetrics and Gynecology—USA

**DOI:** 10.1155/S1064744996000245

**Published:** 1996


Infectious Diseases in Obstetrics and Gynecology 4:105–109 (1996)
© 1996 Wiley-Liss, Inc.

Abstracts From the

### First Annual International Infectious Disease Society for Obstetrics and Gynecology—USA

Branch Meeting, Boulder, Colorado

April 27–28, 1996

Special Section

Received June 4, 1996
Accepted August 8, 1996

### UNUSUAL GYNECOLOGIC MANIFESTATIONS IN HIV+ PATIENTS.

HAMMILL HA.
*Department of Obstetrics and Gynecology, Baylor College of Medicine, Houston, TX.*

In the last 8 years, the gynecologic population followed by a single practitioner in the City of Houston has increased. Now, 600 HIV+ women are being followed. The cervical dysplasia rate in this group has ranged from 40-50%, although the diagnosis of cervical cancer has antedated the HIV diagnosis in only 2 cases. An increasing problem has been recurrent herpetic ulcers. Twelve patients with markedly depressed CD4 counts (< 50) have had positive herpes cultures associated with a failure to respond to oral acyclovir. They have been treated with intravenous acyclovir to arrest the progression of rectovaginal fistulas and erosive ulcers of 2-3 cm in size. Two patients presenting with erosive ulcers were subsequently diagnosed with vulvar cancer, in both cases, after their herpes cultures were negative. In addition, there have been 2 cases of giant condyloma, measuring > 6 cm by 3 cm and requiring surgical correction. One patient was found to have giant (6 cm by 3 cm) molluscum contagiosum mimicking condyloma that occluded the perineum. These atypical presentations heighten the requirement for biopsies and cultures when such patients present for care.

Corresponding author:
Hunter A. Hammill, M.D.
6550 Fannin #701
Houston, TX 77030

### IN VITRO BENZYDAMINE SUSCEPTIBILITY TESTING WITH TIME KILL.

GUNTER J, AULT K, DOZIER D, FARO S.
*University of Kansas Medical Center, Kansas City, KS.*

We evaluated the in vitro activity of benzydamine (BZ) against microorganisms in the vaginal ecosystem with time-kill assays. The killing times for two strains (a wild type and an ATCC) of *Candida albicans, C. glabrata, Streptococcus agalactiae, S. faecalis, Escherichia coli,* and *Gardnerella vaginalis* were determined at 2X MBC, MBC, and 1/2 MBC. If 100% kill did not occur, a 3-$\log_{10}$ decrease in colony forming units (cfu) was considered a sufficient bactericidal response. *C. albicans* and *C. glabrata* had 100% kill at 2X MBC, MBC, and 1/2 MBC. *E. coli* had 100% kill at 2X MBC and MBC, but no decrease in cfu at 1/2 MBC. *S. agalactiae*, both strains, had 100% kill at 2X MBC. At MBC, only the *S. agalactiae* ATCC had a sufficient bactericidal response; both strains had insufficient responses at 1/2 MBC. *G. vaginalis* had 100% kill at 2X MBC and MBC. Only the wild type had a 3-$\log_{10}$ decrease in cfu at 1/2 MBC. *E. faecalis* achieved 100% kill at 2X MBC. At lower levels, the decrease in cfu was variable. A sufficient bactericidal response to BZ occurred at 2X MBC and MBC for *C. albicans, C. glabrata, E. coli,* and *G. vaginalis.* Only *Candida* had 100% kill at 1/2 MBC. *S. agalactiae* and *S. faecalis* achieved bactericidal responses at 2X MBC. These results support the hypothesis that BZ has antibacterial and antimycotic properties warranting further testing against a greater number of vaginal pathogens.

Corresponding author:
Jennifer Gunter, M.D.
Department of Gynecology and Obstetrics
U. of Kansas School of Medicine
3901 Rainbow Boulevard
Kansas City, KS 66160-7316

### ULTRASOUND MARKERS OF FETAL INFECTION.

*BAILÂO LA, BONILLA-MUSOLES F, OSBORNE NG.*

*Universidad de São Paulo, Ribeirão Preto, Brazil (LAB); Universidad de Valencia, Spain (FB-M); Howard University, Washington, DC (NGO).*

Clinically recognizable congenital infections account for only a small proportion of infectious diseases in newborns. However, fetal infections may be one of the most frequent causes of perinatal mortality. The ability to identify ultrasound markers of embryo and fetal infections offers the possibility of identifying etiologic agents, correlating subtle infections with specific antepartum and postpartum syndromes, and reducing the problem of silent infections that result in unexplained adverse pregnancy outcomes. High resolution and color Doppler ultrasound were used to detect the structural and blood-flow changes caused by infection-induced vasculitis of villous stem arteries and of uterine radial and spiral arteries produced by focal lymphocytic and histiocyte infiltration of villi and decidua with resultant necrosis, granular tissue and fibroblastic proliferation, or fibrosis. The placental edema that results from an infection of the villi can, at times, be detected prior to the changes of necrosis and fibrosis of villi. We detected fetal hydrops, fetal and placental pathologic calcification, umbilical-cord edema, and placental thickening in cases of prenatal toxoplasmosis, cytomegalovirus infection, Chagas' diseases, syphilis, and congenital hepatitis. Premature extravillous calcification, pathologic intravillous calcification, and ominous changes in the maternal uterine-artery flow, umbilical-cord blood flow, and fetal middle-cerebral-artery flow are of prognostic significance when associated with infection.

Corresponding author:
Newton G. Osborne, M.D.
2041 Georgia Avenue, NW
Washington, DC 20060

### ETIOLOGY OF NONOXYNOL-9-INDUCED LOWER-GENITAL-TRACT LESIONS.

FORD LC, KESLER VP, HAMMILL HA, KNOBEL DP.
*Department of Obstetrics and Gynecology, University of California-Irvine, Orange, CA (LCF); Lafor Laboratories, Newport Beach, CA (LCF, VPK); Department of Obstetrics and Gynecology, Baylor College of Medicine, Houston, TX (HAH); Surgeon General, SADE, Pretoria, RSA (DPK).*

Nonoxynol-9 is the most commonly used spermicidal agent in the United States. While Nonoxynol-9 has been approved for over 40 years, a recent observation has been made that its frequent use can predispose a patient to the formation of lower-genital-tract lesions. These lesions may become a portal of infection to HIV and other serious pathogens. Using quantitative vaginal cultures, we noted that the daily use of 100 mg of Nonoxynol-9 for 5-15 days caused a destruction of the vaginal lactobacilli and an "altered flora" in which the predominant organisms were the zenteric bacteria, *Streptococcus faecalis*, and abnormally high numbers of anaerobes. These flora changes appear to predispose a patient to vaginal lesions, bacterial vaginosis, and urinary-tract and yeast infections. We postulate that the effects of Nonoxynol-9 on the vaginal flora potentiate the formation of these lesions and, to a lesser extent, direct chemical irritation in a given patient. In patients with these Nonoxynol-9-induced lesions, Inner Confidence™ vaginal suppositories caused both the healing of the observed lesions and the re-establishment of lactobacilli as the predominant flora.

Corresponding author:
Hunter A. Hammill, M.D.
6550 Fannin #701
Houston, TX 77030

### USE OF A NOVEL BACTERIAL LECTIN AS A BIOMARKER IN FEMALE NEOPLASTIC LESIONS.

KAUL A, MARTENS MG, NOWICKI B, KAUL R.
*Department of Obstetrics and Gynecology, University of Minnesota (AK); Department of Obstetrics and Gynecology, Hennepin County Medical Center (MGM); and Minneapolis Medical Research Foundation, Minneapolis, MN (BN, RK).*

Certain bacterial proteins called fimbriae, pili, or adhesins on the bacteria cell surface help the cell to adhere to specific receptors present on the tissue surface and colonize the host. One such bacterial protein is Dr-fimbriae which are present on *Escherichia coli* and associated with infection of the urinary tract. Recently, we reported that Dr-fimbriae recognize a specific tissue receptor on the complement decay-accelerating factor (DAF), a complement regulatory protein. One of the primary functions of the human body defense mechanism is to protect the host from external or foreign cells including cancer cells. This function is performed by the human complement system by selectively killing the foreign or cancer cells by lytic action. The host cells are protected from this toxic or the killing effect by the presence of the host cell surface. The function of these proteins is to inactivate complement, and DAF is an important member of this group. In our studies on tissue samples from the human endometrium, we observed a 400-to 800-fold increase in the expression of DAF/Dr ligand in adenocarcinoma of the endometrium compared with normal tissues. This overexpression of DAF may help cancer cells escape the complement-mediated killing. We also observed a 40- to 50-fold increase in DAF/Dr ligand expression in hyperplastic lesions of the human endometrium. This study may allow us to develop a simple diagnostic aid to detect and follow human cancers using purified Dr-fimbriae. In addition, the use of Dr-fimbriae to inactivate DAF function on cancer cells may provide an entirely new and efficient method to treat cancers by exposing cancer cells to specific drugs or antibodies.

Corresponding author:
Mark G. Martens, M.D.
Department of Obstetrics and Gynecology
Hennepin County Medical Center
701 Park Avenue South
Minneapolis, MN 55415-1829

### AMPLITUDE OF DAILY TEMPERATURE FLUCTUATION WITH RESPECT TO MEAN BODY TEMPERATURE.

MACCATO A, MACCATO M.
*University of California-Irvine, Irvine, CA (AM); Baylor College of Medicine, Houston, TX (MM).*

A study of 97 postpartum patients was performed to analyze the relationship of mean body temperature, the amplitude of daily temperature fluctuations, and the variance of this relation within the sample. The data consisted of body temperature recordings, measured every 4 hours to within two-tenths of a degree Fahrenheit, in postpartum patients under normal clinical conditions. Of the 97 cases, 78 suffered from febrile morbidity, while 19 did not. The patients' hospital stays ranged from 2-18 days, with a median of 5 days. The temperature recordings were grouped into 24-hour segments (6 temperature readings) for each patient. Within each 24-hour segment, 2 parameters were calculated: (1) the mean body temperature T and (2) the difference between the maximum and minimum readings (temperature fluctuation) F. For each patient in the study, a line was fitted to the (T,F) data pairs by minimum square error, yielding a slope coefficient S. For each patient, S served as a measure of the rate of increase in temperature fluctuations per unit increase in mean body temperature. Finally, the mean and standard deviations were computed for the distribution of S values over the patient sample. The entire sample yielded a mean S value of 0.42, with a standard deviation of 0.88. The subsample of 78 febrile patients yielded a mean S value of 0.38, with a standard deviation of 0.65. Both mean S values were statistically significantly distinct from 0 ($P < 0.001$). This study demonstrated a statistically significant positive correlation between mean body temperature and amplitude of daily temperature fluctuations for the entire sample. The study also showed significant variance of this relation within the sample.

Corresponding author:
Maurizio Maccato, M.D.
7400 Fannin, Suite 700
Houston, TX 77054

### INNER CONFIDENCE,™ A FEMALE-CONTROLLED VAGINAL MICROBICIDE CONTRACEPTIVE THAT CAN PREVENT SPREAD OF SEXUALLY TRANSMITTED DISEASES AND REPLENISH NORMAL VAGINAL FLORA.

FORD LC, KESLER VP, HAMMILL HA, LEBHERZ TB, KNOBEL DP.

*Department of Obstetrics and Gynecology, University of California-Irvine, Orange, CA (LCF); Lafor Laboratories, Newport Beach, CA (LCF, VPK); Department of Obstetrics and Gynecology, Baylor College of Medicine, Houston, TX (HAH); Department of Obstetrics and Gynecology, University of California-Los Angeles, Los Angeles, CA (TBL); Surgeon General, SADF, Pretoria, RSA (DPK).*

The World Health Organization, United States National Institutes of Health, and other health authorities have expressed their urgent concerns for the development of a female-controlled, safe, topically active microbicide that could prevent the heterosexual transmission of HIV as well as other sexually transmitted diseases. Inner Confidence™ fulfills all of the efficacy and safety concerns of these organizations. The frequent use of Inner Confidence™ does not cause the vaginal lesions associated with Nonoxynol-9 products. It destroys both the white blood cells and spermatozoa which may act as carriers of infectious organisms and prevents the postcoital rise in vaginal pH. Moreover, it is compatible with latex condoms. Intravaginally, Inner Confidence™ kills *Gardnerella vaginalis, Neisseria gonorrhoeae*, the pyogenic cocci, the enterics, *Candida*, *Chlamydia trachomatis*, *Trichomonas vaginalis*, herpes simplex 2, HIV, and other pathogens for 8 hours. After the antimicrobial concentrations ebb, hydrogen peroxide and microcidin-producing, micro-encapsulated lactobacilli are released which replenishes the normal vaginal flora and appears to prevent genital-tract lesions.

Corresponding author:
Hunter A. Hammill, M.D.
6550 Fannin #701
Houston, TX 77030

### BACTERIAL VAGINITIS AND TRICHOMONIASIS–SYMBIOSIS OR PROGRESSION.

MARTENS MG, EL-ZAARTARI M.

*Department of Obstetrics and Gynecology, University of Minnesota and Hennepin County Medical Center, Minneapolis, MN.*

*Trichomonas vaginalis* (TV) is one of three major causes of vaginitis in women. The association of TV with bacterial vaginosis (BV) has been reported, but rarely analyzed. Before metronidazole, TV was treated by maintaining a normal pH range (3.8-4.2) of the vagina to decrease TV growth, indicating the important role of the vaginal pH in TV survival. Menstrual blood, semen, and vaginal discharges, pathogens, and syndromes such as BV transform the pH to a less acidic level (>4.5) favoring TV growth. To determine an association of BV in the pathogenicity of TV, we screened for both infections. Presenting to the gynecology clinic for nonannual examinations were 72 nonpregnant, premenopausal women, of whom 40 had complaints of vaginal discharge (symptomatic) and 32 had no complaints (asymptomatic). Each patient had a vaginal sample examined for pH, wet preparation, 10% KOH, and Gram's stain smear. A patient was excluded if she had a positive screening test for *Chlamydia trachomatis* or *Neisseria gonorrhoeae* or if she had hyphae or yeast cells on the wet preparation. A TV culture using Diagmond's medium was also performed. The Gram's stain criteria for BV diagnosis described by Nugent et al. was followed. Twenty-seven (37%) patients were diagnosed with TV; 29 (40%) were noted to have met the Gram's stain criteria for BV (pH >4.5); 12 (16%) were considered normal; and 4 (7%) were excluded. Of the 27 TV patients, 21 (78%) were symptomatic and 6 (22%) were asymptomatic. Twenty-one of the 24 (88%) TV patients were also found to have a score of 7 according to the Gram's stain BV criteria. In asymptomatic patients, 4 of 6 with elevated pHs and WBCs on the wet preparations were positive for TV compared with 10 with elevated pHs, but no WBCs (P=0.008). The TV and BV patients appeared to share a similar environment, diverging only in the area of increased WBCs for the TV patients and increased clue cells for the BV patients. TV may require an altered environment for infection that is augmented or supplied by factors similar to those seen with BV. Therefore, an asymptomatic patient with an elevated pH and WBCs seen on the wet preparation should have a thorough examination, if not a culture for TV.

Corresponding author:
Mark G. Martens, M.D.
Department of Obstetrics and Gynecology
Hennepin County Medical Center
701 Park Avenue South
Minneapolis, MN 55415-1829

### URINE TESTING WITH LIGASE CHAIN REACTION FOR DETECTION OF *CHLAMYDIA TRACHOMATIS*.

*AULT K, GUNTER J, JOACHIMS M, DOZIER D, WITT J.*
*University of Kansas Medical Center, Kansas City, KS.*

We sought to determine the sensitivity of urine testing using ligase chain reaction (LCR) (Abbott Diagnostics Division, Abbott Park, IL) for the detection of *Chlamydia trachomatis* (CT) infection. Four hundred eight women were enrolled from 2 family planning sites. Nearly all patients were asymptomatic. A urine sample was obtained from each patient. After a pap smear was obtained, one swab was collected for the Gen-Probe and another for a CT culture. The culture was carried out with shell vials of BCMK cells and stained at 48-72 hours with FITC-labelled monoclonal antibodies. The Gen-Probe and LCR tests were performed according to the manufacturers' instructions. A specimen was considered a "true positive" if the culture was positive or if both nonculture tests were positive and the culture was negative. Further discrepancies were resolved by an alternative LCR test for DNA encoding for the major outer membrane protein (MOMP) of CT. "True positive" results were obtained in 16 patients. All 3 tests were negative in 385 patients. Four specimens were false-negative LCRs. Three specimens were positive by only LCR and confirmed by the LCR-MOMP. The prevalence was 5.6%. After the discrepant results were resolved, the LCR sensitivity was 82.6% and specificity was 100%. The positive predictive value was 100% and negative predictive value was 99.0%. This urine-based CT screening technique has a similar sensitivity to those of other nonculture tests. It has allowed us to expand our screening efforts to women without access to pelvic examinations.

Corresponding author:
Kevin A. Ault, Ph.D.
Department of Gynecology and Obstetrics
U. of Kansas School of Medicine
3901 Rainbow Boulevard
Kansas City, KS 66160-7316

### INTERRELATIONSHIPS WITHIN THE BACTERIAL FLORA OF THE FEMALE GENITAL TRACT.

*CARSON HJ, LAPOINT PG, MONIF GRG.*
*Department of Pathology, Resurrection Medical Center, Chicago, IL (HJC); Department of Obstetrics and Gynecology, Creighton University School of Medicine, Omaha NE (PGL, GRGM).*

An analysis of 239 consecutive vaginal swabs using the compatibility profile technique revealed that only 2 bacteria have the ability to be sole isolates and, as such, to be candidates as major aerobic regulators of the bacterial flora of the female genital tract. Compatibility profiles of *Lactobacillus* and *Gardnerella vaginalis* have shown that these organisms share compatibility profiling for the majority of the normal bacterial constituents of the female genital tract. Dominance disruption appears to come from the addition of compatible co-isolates and disruption of numerical dominance. These phenomena appear to be the keys to re-regulation of the bacterial flora of the female genital tract. Lactobacilli appear to be the major regulators of both *G. vaginalis* and anaerobic bacteria. When additional organisms are added to the bacterial flora, they may add to or partially negate the *Lactobacillus* inhibitory influence on the bacterial flora of the female genital tract. Inhibitor interrelationships appear to exist between coagulase-negative staphylococci and *Staphylococcus aureus* and the group-B streptococci and other â-hemolytic streptococci. Facilitating interrelationships appear to exist between *S. aureus* and the group-B streptococci and selected *Enterobacteriaceae*.

Corresponding author:
Gilles R. G. Monif, M.D.
Department of Obstetrics and Gynecology
Creighton University School of Medicine
601 North 30th Street
Omaha, NE 68131

